# Artemisinin resistance and malaria elimination: Where are we now?

**DOI:** 10.3389/fphar.2022.876282

**Published:** 2022-09-23

**Authors:** Borimas Hanboonkunupakarn, Joel Tarning, Sasithon Pukrittayakamee, Kesinee Chotivanich

**Affiliations:** ^1^ Department of Clinical Tropical Medicine, Faculty of Tropical Medicine, Mahidol University, Bangkok, Thailand; ^2^ Mahidol Oxford Tropical Medicine Research Unit, Faculty of Tropical Medicine, Mahidol University, Bangkok, Thailand; ^3^ Centre for Tropical Medicine and Global Health, Nuffield Department of Medicine, University of Oxford, Oxford, United Kingdom; ^4^ The Royal Society of Thailand, Bangkok, Thailand

**Keywords:** *Plasmodium falciparum*, drug resistance, pharmacokinetics, pharmacodynamic, mechanism of resistance, artemisinin-based combination therapies

## Abstract

The emergence of artemisinin resistance is a major obstacle to the global malaria eradication/elimination programs. Artemisinin is a very fast-acting antimalarial drug and is the most important drug in the treatment of severe and uncomplicated malaria. For the treatment of acute uncomplicated falciparum malaria, artemisinin derivatives are combined with long half-life partner drugs and widely used as artemisinin-based combination therapies (ACTs). Some ACTs have shown decreased efficacy in the Southeast Asian region. Fortunately, artemisinin has an excellent safety profile and resistant infections can still be treated successfully by modifying the ACT. This review describes the pharmacological properties of ACTs, mechanisms of artemisinin resistance and the potential changes needed in the treatment regimens to overcome resistance. The suggested ACT modifications are extension of the duration of the ACT course, alternating use of different ACT regimens, and addition of another antimalarial drug to the standard ACTs (Triple-ACT). Furthermore, a malaria vaccine (e.g., RTS,S vaccine) could be added to mass drug administration (MDA) campaigns to enhance the treatment efficacy and to prevent further artemisinin resistance development. This review concludes that artemisinin remains the most important antimalarial drug, despite the development of drug-resistant falciparum malaria.

## Introduction

Artemisinin remains the most important antimalarial drug despite the development of partial drug resistant *Plasmodium falciparum* parasites in Southeast Asia (Dondorp et al., 2009; Ashley et al., 2014), and emerging partial resistance in East-Africa (Uwimana et al., 2020; Balikagala et al., 2021; Uwimana et al., 2021). It is the fastest acting antimalarial drug available in the market, and plays a key role in the treatment of uncomplicated and severe malaria. Artemisinin-based combination therapy (ACT), given orally for 3 days, is the first-line therapy recommended in the treatment of uncomplicated *P. falciparum* malaria. Parenteral artesunate is the first line treatment for severe malaria, and should be continued until the patient is well enough to receive oral medication. While the world is aiming to eliminate malaria, artemisinin resistance threatens this objective. This review aims to describe the pharmacological properties of ACTs, the mechanism of artemisinin resistance and the potential changes needed in the treatment regimens to fight resistance.

## Pharmacokinetic-pharmacodynamic properties of ACTs

**FIGURE 1:**
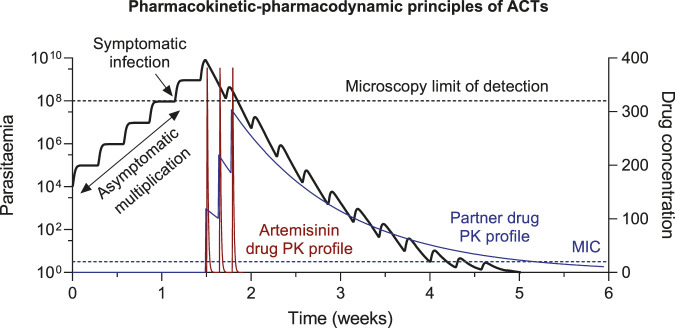
Pharmacokinetic-pharmacodynamic (PK-PD) principles of ACT treatment. The minimal inhibitory concentrations (MIC) is the lowest antimalarial drug concentration that will inhibit the visible growth of the parasites, resulting in parasite elimination at drug concentrations >MIC and parasite growth at drug concentrations <MIC.

Artemisinin is a short acting but very potent antimalarial drug. It has a short terminal elimination half-life of approximately 1–3 h, resulting in undetectable drug levels within 10 h of drug administration (Tarning et al., 2012; Kloprogge et al., 2015). This pharmacokinetic property demands daily dosing to achieve effective treatment. However, a relatively high degree of recrudescence was seen with short courses of daily monotherapy (<7 days), due to artemisinin-induced dormancy in *P. falciparum* infections (Peatey et al., 2021). Artemisinin is no longer recommended to be used as monotherapy, except for parenteral treatment of severe malaria. In ACTs, artemisinin is responsible for eliminating the majority of the parasite biomass during the first days of treatment, while residual partner drug concentrations kill the remaining parasites to prevent recrudescence (Figure 1). The partner drug concentrations must be maintained above the minimal inhibitory concentration (MIC) until the infection has been cleared for successful chemotherapy. If the drug concentrations fall below the MIC value before the infection is cleared, parasites could multiply and result in a recrudescent infection (Figure 1).

This time period, after drug administration, where residual drug concentrations are maintained above the MIC value is also referred to as post-treatment prophylaxis. Novel infections emerging from the liver during this time-period will be eliminated and therefore prevent reinfections to be established. The suppression of novel infections associated with long-lasting ACTs are utilized during the prevention of malaria (i.e., prophylactic treatment, seasonal malaria chemotherapy (SMC), intermittent preventive treatment (IPT)). The duration of post-treatment prophylactic effect depends on both the terminal elimination half-life of the drug and the susceptibility of the parasite to the drug, which determines the MIC. ACTs containing a partner drug with a long terminal elimination half-life can be used effectively in the prevention of malaria. The most promising combination is dihydroartemisinin-piperaquine, due to the long biological half-life of piperaquine (20–30 days), which allows monthly treatment to prevent malaria (Hoglund et al., 2017; Chotsiri et al., 2019).

Parasite drug resistance is the result of genetic changes that confers reduced drug susceptibility. This allows a mutated parasite to multiply when exposed to otherwise suppressive drug concentrations. Such a survival advantage, compared to wildtype parasites, would quickly be selected for during high drug pressure in a region, allowing the drug resistant strain to spread. Genetic mutations are by definition random events, and the absolute number of genetic mutations during an infection is therefore expected to increase with increasing parasitemia. Thus, *de novo* drug resistance is more likely to arise in patients with high parasitemia. The probability of drug resistance development would therefore be higher in patients with hyperparasitemia (>10^12^ parasites), compared to patients with uncomplicated acute symptomatic infections (10^8^–10^12^ parasites), compared to patients with asymptomatic infections (<10^8^ parasites). When/if such an event occurs, exposure to another drug with a different mechanism of action would eliminate the mutated parasite and prevent the drug resistant parasite to establish a patent infection. This is the main reason why combination therapies prevent drug resistance development. The ideal combination of drugs would be two (or more) highly potent compounds with different mechanisms of action, and matching pharmacokinetic properties (i.e., similar terminal elimination half-lives). This would allow drug concentrations to be maintained above the MIC value for a similar duration of time in order to prevent emerging resistant parasites to establish a patent infection.

The artemisinin class of drugs (i.e., artesunate, artemether, and dihydroartemisinin) is the most potent and rapidly acting antimalarials on the market. Artemisinin commonly result in approximately 10^3^–10^4^-fold reduction in parasitemia per parasite life-cycle of 48 h, while the marketed ACT partner drug is less potent, resulting in approximately 10^1^–10^3^-fold reduction in parasitemia per parasite life-cycle of 48 h. It would be highly advantageous with novel partner drugs that are more potent than the currently available antimalarials. This rapid reduction in parasitemia associated with ACT reduces the likelihood of resistance development. However, decades of preceding artemisinin-based monotherapy resulted in the development of artemisinin-resistant parasites in Cambodia, which is now widespread throughout mainland Southeast Asia, resulting in a slower parasite clearance of *P. falciparum* Kelch13-mutated parasites compared to wildtype parasites (Ariey et al., 2014; Ashley et al., 2014; Kagoro et al., 2022; Zhu et al., 2022). Artemisinin resistance has now also emerged in India (Chakrabarti et al., 2019; Das et al., 2019), in South America (Mathieu et al., 2021), and on the African continent in Uganda and Rwanda (Uwimana et al., 2020; Balikagala et al., 2021; Uwimana et al., 2021). Artemisinin-resistant infections are defined as patients showing a parasite clearance half-life above 5 h, at standard dosing (i.e., <10^3^-fold reduction in parasitemia per life-cycle) (Dondorp et al., 2009). This results in a substantially higher parasite biomass to be eliminated by the partner drug in ACTs, and as a consequence, multi-drug resistant parasites have now developed in Southeast Asia with reduced drug susceptibility towards both artemisinin and its partner drug (Amaratunga et al., 2016). We and others have suggested to use longer duration ACT courses and triple ACTs (TACTs) in the fight against multi-drug resistant malaria. The additional third drug would be selected to match the pharmacokinetic properties of the existing ACT partner drug (e.g., dihydroartemisinin-piperaquine + mefloquine). TACTs have shown excellent treatment efficacy in regions of multi-drug resistant malaria, and they are likely to protect against further drug resistance development (van der Pluijm et al., 2020; Peto et al., 2022).

**FIGURE 2:**
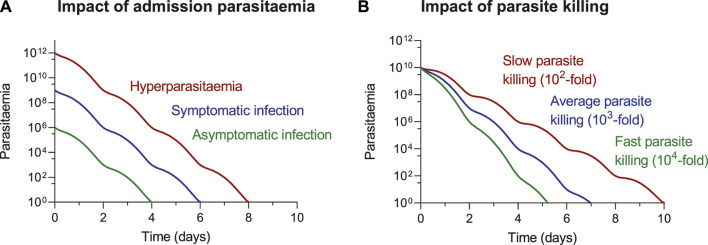
ACT-related elimination of a malaria infection, illustrating the impact of **(A)** starting parasitaemia and **(B)** rate of parasite decline on time to clearing an infection. The impact of parasitaemia **(A)** is illustrated assuming a 10^3^-fold reduction in parasitaemia every parasite lifecycle of 48 h. The impact of parasite decline **(B)** is illustrated assuming an admission parasitaemia of 10^10^ parasites.

ACT-related elimination of a malaria infection results in an exponential decay of parasites. The time it takes to clear an infection is dependent on two variables (1) starting parasitemia and (2) rate of decline (Figure 2). Thus, it takes a longer time to clear an infection with high admission parasitemia (hyperparasitemia) compared to an infection with low admission parasitemia (asymptomatic). Similarly, it takes a longer time to clear resistant infections (slower rate of decline) compared to drug susceptible infections (higher rate of decline). Therefore, it is substantially higher risk for resistant development and more difficult to treat symptomatic patients compared with asymptomatic patients. Increased drug pressure in malaria elimination campaigns, resulting from mass-drug-administrations or screen-and-treat strategies, might not increase the risk of resistance development, when using high-quality drugs administered according to standard guidelines. Most individuals receiving these treatments would be asymptomatic patients, with very low levels of parasitemia. Their infections could be treated relatively quick. The low starting parasitemia and the relatively short duration of infection, would make these individuals far less likely to develop antimalarial drug resistance.

## Mechanisms of artemisinin resistance

Artemisinin resistance was first reported in Cambodia (Dondorp et al., 2009; Ashley et al., 2014), and have now spread throughout the Greater Mekong Subregion (GMS) (Imwong et al., 2020). Clinical artemisinin resistance can be detected by several *in vitro* susceptibility tests, i.e., the trophozoite inhibition maturation assay (TMI) with an IC_50_ value >5 ng/ml (Chotivanich et al., 2014); and the ring survival assay (RSA) with a parasite survival rate ≥1% (Witkowski et al., 2013a; Witkowski et al., 2013b; Davis et al., 2020). Molecular studies have demonstrated that mutations in the *P. falciparum* Kelch 13 (PfK13) gene are associated with a prolonged clinical parasite clearance (Ariey et al., 2014). The PfK13 protein is involved in the endocytosis of hemoglobin, which is required for parasite growth in red cells. The strong association between PfK13 mutant alleles, *in vitro* parasite survival rate (TMI and RSA), and *in vivo* parasite clearance half-life indicate that PfK13 mutations are an important molecular marker of artemisinin resistance (Wang et al., 2015; Thuy-Nhien et al., 2017; Sá et al., 2018; Chhibber-Goel and Sharma, 2019; Das et al., 2019; Zhang et al., 2019; Miotto et al., 2020; Siddiqui et al., 2020). PfK13 contains six β-propeller Kelch domains and mutations in this region, particularly the C580Y mutation, has been used in the surveillance of artemisinin resistance in the GMS. However, artemisinin resistance (i.e., prolonged parasites clearance half-life) has also been reported without PfK13 mutations in clinical isolates from Cambodia (Mukherjee et al., 2017) and India (Das et al., 2021).

The following mechanisms have been proposed to explain the development of artemisinin resistance; 1) reduced ring stage drug susceptible compared to the mature stages (Wang et al., 2018; Intharabut et al., 2019), 2) enhanced adaptive responses against oxidative stress and protein damage (Mok et al., 2015), 3) lower levels of ubiquitinated proteins, resulting in enhanced cell stress response and delayed cell death (Dogovski et al., 2015; Tilley et al., 2016), 4) an elevation of a lipid phosphatidylinositol-3-phosphate (PI3P) as a result of the reduced binding between *P. falciparum* phosphatidylinositol-3-kinase (PfPI3K) and mutated PfK13 (Mbengue et al., 2015; Suresh and Haldar, 2018), 5) other unknown mechanisms protecting the infected red cell from proteopathy (Rocamora et al., 2018), and 6) better adherence to host receptors and remodeling of the infected red cell membrane by vesicle amplification to avoid splenic removal (Bhattacharjee et al., 2018).

Dormant *P. falciparum* parasites after artemisinin exposure have been reported *in vitro* (Teuscher et al., 2010) and *in vivo* (Peatey et al., 2021). This has raised the concern over the potential association of the artemisinin-induced dormant parasites and the development of drug resistance as found in other microbes (Lewis, 2007). Besides, a study on *P. falciparum* infection in the *Aotus* monkey model showed little effect of PfK13 mutations in the recrudescent frequency after artemisinin treatment (Sa et a., 2018). This could be a result of dormant parasites or the differences between humans and monkeys in host-parasite relationships. The possibility of artemisinin-induced dormancy emphasizes the importance of partner drugs in ACTs.

Immunity is also suggested to play important roles in the emergence and spreading of antimalarial resistance. Non-immune people almost always become ill when they are first infected with malaria. Therefore, young children in endemic areas are the main group that suffers from the infection; many develop severe malaria and die. With repeated exposure, people can acquire immunity against malaria although the immunity is slowly developed and wanes after a few years if not constantly exposed to the infection. This naturally acquired antibody-mediated immunity can lower parasitemia due to its effect on blood-stage parasites (Marsh and Kinyanjui, 2006), and reduce transmission due to its effect on gametocytes and sporozoites (John et al., 2005 and John et al., 2008; Bousema and Drakeley, 2011). Therefore, the risk of severe infection and death are lower in older children and adults in malaria endemic areas. Interestingly, it has been noticed that although antimalarial resistance can be found worldwide, the origin of resistance is typically in low transmission areas. Chloroquine resistance in *Plasmodium falciparum* appeared in Thailand (Harinasuta et al., 1962) just 2 years before Colombia (Moore and Lanier, 1961) during late 1950s, both of which were low transmission areas. The resistance continuously spread across Southeast Asia to India and arrived Africa in 1978 (Campbell et al., 1979; Fogh et al., 1979). This route of spreading the resistant genotype was later confirmed by genetic microsatellite analysis (Wootton et al., 2002). Decades later, the history has repeated itself with sulfadoxine-pyrimethamine resistance (Roper et al., 2004) and now with artemisinin resistance.

Suggested explanations of this phenomenon have been proposed recently (Ataide et al., 2017; Whitlock et al., 2021). A multinational study of artemisinin resistance in Southeast Asia demonstrated that faster parasite clearance time was associated with higher immunity levels, suggesting that patients with low immunity are more likely to develop, harbor and transmit mutant parasites (Ataide et al., 2017). A multiscale, agent-based model by Whitlock et al. also provided supportive findings, showing that high host immunity, as acquired in high transmission setting, slowed the evolution of resistance. However, once the resistance becomes common in the parasite population, it will be maintained at a high prevalence (Whitlock et al., 2021).

With global malaria elimination programs, malaria transmission and thus host immunity will be lower in many areas, resulting in greater risk of emergence and expansion of artemisinin-resistant *P. falciparum* infections.

Multi-drug resistant parasites have now emerged as a consequence of widespread artemisinin resistance, resulting in high failure rates of the ACT dihydroartemisinin-piperaquine in western Cambodia and nearby regions such as northeast Thailand and southern Vietnam (Amato et al., 2017; Witkowski et al., 2017; van der Pluijm et al., 2019). In India, longitudinal *Plasmodium falciparum* isolates from across the country showed decreased *in vitro* artemisinin-sensitivity as early as 2012 (Chakrabarti et al., 2019) and declining clinical efficacy of artesunate-sulfadoxine-pyrimethamine has been reported (Mishra et al., 2014; Das et al., 2021). Artemisinin is the most important antimalarial drug and remains a critical tool for malaria elimination and eradication programs worldwide. It is crucial to understand where resistance occurs geographically and the different resistance mechanisms and parasite-host interactions associated with reduced drug susceptibility. Clinical studies and epidemiological surveillance are needed for the containment of artemisinin resistance. Identifying the clinical efficacy of available drugs and combinations, and developing new antimalarial compounds with novel mechanisms of action is needed urgently.

## Potential modifications of artemisinin-based treatments for increased efficacy

Although resistance to artemisinin and its derivatives has emerged among *P. falciparum* in various regions (Ashley et al., 2014), ACTs are still the main stay of *falciparum* malaria treatment. Several researchers have explored various ways to improve the use of artemisinin to maximize its benefits. Modification of existing antimalarial regimens has been considered as the quickest way to improve the treatment outcome. Potential modifications include a prolonged ACT course, alternate use of different ACT regimens, and triple ACTs. Combining a malaria vaccine and an ACT in mass drug administration (MDA) campaigns in low transmission settings have been suggested to accelerate the elimination of *falciparum* malaria.

### Prolonged treatment course

Several studies have suggested that extending ACTs to 5–6 days could increase the effectiveness of ACTs. While the 3-day ACTs are the first-line therapy recommended by the WHO in treating uncomplicated *falciparum* malaria, parasites are potentially exposed to artemisinin for only one full parasite life cycle during the treatment course (White, 1997), which might not be enough for effective treatment of resistant parasites (White, 1997; Walker and Sullivan, 2017). Pharmacokinetic modelling of artemether-lumefantrine showed substantially increased exposure with a prolonged duration of treatment (Tarning et al., 2009). Pharmacological modelling, based on dihydroartemisinin-piperaquine, suggested that ACT effectiveness in the treatment of artemisinin resistant infections, could be improved if treatment duration was extended from 3 to 5 days (Kay et al., 2015). Results from a murine malaria model suggested that artemisinin’s cytocidal activity was enhanced when extending the treatment duration to cover 3 asexual life-cycles compared to 1 life-cycle with similar total dose (Walker and Sullivan, 2017). While these results were promising, it raised concerns related to the safety of prolonged exposure to long half-life partner drugs. Recently, a 5-day regimen of artemether-lumefantrine was demonstrated to be well tolerated and safe when treating uncomplicated *falciparum* malaria in pregnant and non-pregnant women in Africa (Onyamboko et al., 2020) and in adult non-pregnant patients in an area of emerging artemisinin resistance in Myanmar (Tun et al., 2018). Additional information on other ACT regimens is needed to support this modification. Furthermore, in a real-life setting, prolonged treatment courses are likely to result in poorer patient compliance, which is needed to be evaluated before implementation.

Increased frequency of ACT administration has been explored and debated. A mathematical modelling approach predicted that increasing the frequency of artesunate administration to twice daily would be beneficial in treating artemisinin-resistant parasites (Saralamba et al., 2011). However, data from a randomized clinical trial in uncomplicated malaria showed that parasite clearance half-life was not statistically accelerated when using twice-daily artesunate (Das et al., 2013). This discrepancy between clinical data and the model prediction was suggested to be a potential reduction of artemisinin sensitivity among other stages of parasites besides the ring stage activity that was the main driver of artemisinin resistance in the model. Additional clinical data are needed to support this practice.

### The alternate use of different antimalarial regimens

There is evidence suggesting that parasites can regain the sensitivity to an antimalarial after the drug pressure has been lifted for some time. In 1993, Malawi withdrew chloroquine and started to use sulfadoxine-pyrimethamine for the treatment of malaria due to the poor clinical efficacy of chloroquine at that time. The molecular marker of chloroquine resistance in *falciparum* malaria, Pf CRT, gradually declined after the withdrawal and was undetectable by 2001. In 2005, a randomized control trial in more than 200 children with uncomplicated *falciparum* malaria in Malawi was performed to compare the efficacy between chloroquine and sulfadoxine–pyrimethamine. The study revealed that the efficacy of chloroquine was almost 100%, while the efficacy of sulfadoxine-pyrimethamine was below 50% (Laufer et al., 2006).

A similar situation was found in Cambodia with artesunate-mefloquine (ASMQ) (Leang et al., 2013). ASMQ was the first-line ACT used in Cambodia for the treatment of drug-resistance *P. falciparum* malaria. The non-fixed co-blistered formulation was introduced in 2000 and used at a national scale until 2008. Overall, country-wide therapeutic efficacy studies (TES) during 2001–2004 showed >90% treatment efficacy, i.e., 28-day PCR-corrected rates of adequate clinical and parasitological response (ACPR) (Denis et al., 2006). However, 28-day ACPR in Western Cambodia, the epicentre of antimalarial drug resistance, showed borderline results in 2001 and nearly 20% failure rates of ASMQ were reported from this region in 2004 (Rogers et al., 2009). This resulted in the replacement of ASMQ with dihydroartemisinin-piperaquine (DP) as first-line therapy in 2008. However, already in 2010, poor clinical efficacy of DP was reported in Western Cambodia (Leang et al., 2015). In the same year, a small study by Leang and coworkers (2013) demonstrated an improvement of ASMQ in Cambodia only after 2 years of withdrawal of the combination from the country. On the other hand, a randomized controlled trial comparing DP, ASMQ and a 4-day artemether-lumefantrine (AL) regimen in pregnant women with uncomplicated malaria on the Thailand-Myanmar border demonstrated that DP was the only drug that had satisfactory efficacy for *P. falciparum* malaria in this area (Saito et al., 2021). These data support the approach of alternate use of different antimalarial combination therapies. Further studies are needed to propose an optimal drug rotation schedule and the timing of each rotation, and to evaluate if it is programmatically feasible to rotate ACT regimens.

In many Sub-Saharan countries artesunate-amodiaquine and artemether-lumefantrine are the main recommended ACTs for treating uncomplicated *falciparum* malaria. Several alternative ACT regimens are also available. A recent systematic review and meta-analysis on the efficacy of ACTs used in Sub Saharan Africa from 2010 to 2020 reports an overall high success rate in the treatment of malaria using artesunate-amodiaquine, artemether-lumefantrine, and dihydroartemisinin-piperaquine in this region (Marwa et al., 2022). With the emergence of partial artemisinin-resistant *P. falciparum* in east Africa (Uwimana et al., 2020; Balikagala et al., 2021; Uwimana et al., 2021), regular surveillance of ACTs efficacy is critical and the availability of several ACTs in the region would be beneficial if properly used.

### Triple artemisinin-based combination therapy

Combination therapies have become a common practice in the treatment of various diseases, both infectious and non-infectious. In 2013, more than 10,000 clinical trials related to the investigation of combination therapies were registered in the US alone (Rationalizing combination therapies, 2017). There are several rationales behind this approach. Using multiple drugs with different mechanisms of action can increase treatment efficacy by targeting multiple components of the organism and reduce the dose of the individual drugs for a more acceptable side effect profile. There might also be pharmacological interactions between the drugs resulting in the additive or synergistic treatment effects. For infectious diseases, combination therapy has shown to also delay or prevent the development of drug-resistant microbes (Mouton, 1999).

The primary aim of the design of ACTs were to increase treatment efficacy and prevent the development of artemisinin resistant *P. falciparum* parasites. Triple artemisinin-based combination therapies (TACTs) were suggested to treat multi-drug resistant falciparum malaria treatment, by adding a second slowly eliminated partner drug to a standard ACT. Mefloquine and amodiaquine were proposed to be paired with piperaquine and lumefantrine, respectively, based on their potentially counterbalancing resistance mechanisms and similar pharmacokinetic profiles. A study in healthy adult Thai volunteers showed that DP + mefloquine was safe and well tolerated (Hanboonkunupakarn et al., 2019).

A multicentre randomised controlled trial involving 1,100 patients with uncomplicated *P. falciparum* malaria from 8 countries demonstrated that DP + mefloquine and AL + amodiaquine were safe, well-tolerated and had excellent efficacy compared to their corresponding standard ACT. As all of these drugs are already in the market, these TACTs can be readily available treatment options for uncomplicated *falciparum* malaria, especially in the GMS where artemisinin and partner-drug resistance are increasingly reported. The use of TACTs in areas where drug resistance has not yet been found might also delay the emergence of drug resistance (van der Pluijm et al., 2020). However, transient increased serum creatinine was found in patients treated with AL + amodiaquine compared to that of other treatment regimens. Additional studies on the renal safety of this TACT is needed to clarify the finding in order to support the use of this combination therapy.

### ACT in combination with malaria vaccine in MDA efforts to eliminate malaria

*Plasmodium falciparum* transmission in Southeast Asia has markedly decreased over the last 2 decades (World Health Organization, 2021). However, the current mainstay of the malaria control program (i.e., vector control, early diagnosis, and effective antimalarial treatment) are unlikely to eliminate *P. falciparum* malaria without an additional strategy to clear the infectious reservoir in asymptomatic populations, especially in the GMS. Mass drug administration (MDA) has been considered as a potential intervention to reduce the infectious reservoir in asymptomatic populations and thus accelerate malaria elimination (Poirot et al., 2013; World Health Organization, 2015).

A cluster-randomised trial using 3 monthly rounds of 3-day DP MDA in GMS where artemisinin-resistant *P. falciparum* is prevalent has shown promising but transient results. *P. falciparum* prevalence and incidence was significantly decreased in MDA villages compared to control villages. Around 90% of asymptomatic *P. falciparum* infections, including those with artemisinin-resistant and piperaquine-resistant parasites, were cleared after MDA. However, the infections returned during the follow-up period, but lower than baseline levels, due to the spread of residual infections and the reintroduction of parasites from other areas (von Seidlein et al., 2019). This is in fact a predictable result as the protection from drug level and immunity wanes over time. Therefore, the effectiveness of MDAs depends on good community engagement, continuous support for village health workers, proper drug selection for the MDA area, and control of malaria reintroduction.

Mathematical models suggested that mass implementation of a malaria vaccine, even one with a temporary effect, in combination with basic malaria control measures could enhance the interruption of parasite transmission. Thus, combining mass vaccination and mass drug administration could lead to a permanent interruption of transmission (Tun et al., 2017). RTS,S/AS01 is currently the only malaria vaccine that has passed a phase 3 trial and it has been used in immunization programs for African children in limited areas under the evaluation of WHO. This vaccine provides short-term partial protection against uncomplicated and severe malaria (RTS,S Clinical Trials Partnership et al., 2011; RTS,S Clinical Trials Partnership et al., 2012). Interestingly, the vaccine showed more protective effect in children living in lower transmission settings than in higher transmission settings (RTS,S Clinical Trials Partnership, 2015). A recent study in healthy adult Thai volunteers demonstrated that the RTS,S/AS01 vaccine was safe and immunogenic in this population and not affected by the concomitant administration of DP and primaquine, which are antimalarials commonly used in MDA (von Seidlein et al., 2020). These data support the use of ACTs in combination with malaria vaccine RTS,S/AS01 in MDA to accelerate *P. falciparum* elimination in Asia. However, further large-scale evaluations in the GMS are needed.

## Conclusion

This review provides an overview of artemisinin resistance, focusing both on clinical and laboratory aspects. The current efficacy of artemisinin-based combination treatments in areas of multi-drug resistant malaria and the widespread emergence of artemisinin resistance, emphasize the importance of global malaria eradication/elimination programs. In-depth understanding of the pharmacological properties of antimalarials and resistant mechanisms is crucial in the use of modified ACTs in the fight against malaria. The potential modifications included three methods to improve the efficacy; 1) prolonged ACT treatment course, which is easy to apply with existing regimens but concerns have been raised on possible side effects of the long half-life drugs and patient compliance; 2) alternate sequential use of different ACT regimens in a region, which can also be applied with existing regimens but additional information are needed on optimal drug rotation timing; and 3) triple ACTs, which has additional benefits over the first two methods of increased efficacy and the ability to delay the development of drug resistance but drug-drug interaction data are needed to support the global implementation of TACTs. Another potential use of ACTs is in combination with malaria vaccine in MDA efforts to accelerate the elimination of malaria in low transmission setting.
